# Exploring “patient-centered” hospitals: a systematic review to understand change

**DOI:** 10.1186/s12913-017-2306-0

**Published:** 2017-05-22

**Authors:** Irene Gabutti, Daniele Mascia, Americo Cicchetti

**Affiliations:** 1 Department of management, Università Cattolica del Sacro Cuore, Largo Francesco Vito, 1, 00168 Rome, Italy; 2 Department of Management, University of Bologna, Bologna, Italy; 3 Department of management, Università Cattolica del Sacro Cuore, Rome, 00168 RM Italy

**Keywords:** Literature review, Hospitals, Organizational change, Patient centered care

## Abstract

**Background:**

The healthcare scenario in developed countries is changing deeply: patients, who are frequently affected by multi-pathological chronic conditions, have risen their expectations. Simultaneously, there exist dramatic financial pressures which require healthcare organizations to provide more and better services with equal (or decreasing) resources. In response to these challenges, hospitals are facing radical transformations by bridging, redesigning and engaging their organization and staff.

**Methods:**

This study has the ambitious aim to shed light and clearly label the trends of change hospitals are enhancing in developed economies, in order to fully understand the presence of common trends and which organizational models and features are inspiring the most innovative organizations. The purpose is to make stock of what is known in the field of hospital organization about how hospitals are changing, as well as of how such change may be implemented effectively through managerial tools. To do so the methodology adopted integrates a systematic literature review to a wider engaged research approach.

**Results:**

Evidence suggests that the three main pillars of change of the system are given by the progressive patient care model, the patient-centered approach and the lean approach. However, there emerge a number of gaps in what is known about how to exploit drivers of change and their effects.

**Conclusions:**

This study confirms that efforts in literature are concentrated in analyzing circumscribed experiences in the implementation of new models and approaches, failing therefore to extend the analysis at the organizational and inter-organizational level in order to legitimately draw consequences to be generalized. There seem to be a number of “gaps” in what is known about how to exploit drivers of change and their effects, suggesting that the research approach privileged till now fails in providing a clear guidance to policy makers and to organizations’ management on how to concretely and effectively implement new organizational models.

**Electronic supplementary material:**

The online version of this article (doi:10.1186/s12913-017-2306-0) contains supplementary material, which is available to authorized users.

## Introduction

The demand of healthcare services in developed countries has changed deeply. The population is ageing and is therefore characterized by multi-pathological chronic conditions. Patients, however, are indeed more informed about their rights and, consequently, expectations from the public health care system seem to have risen considerably. On the other hand, however, there exist dramatic financial pressures which require healthcare organizations to provide more and better services with equal (if not decreasing) resources. Therefore it is clear that many features of healthcare systems are doomed to change if the whole system is to remain sustainable in time. In particular, it has become necessary to pursue outstanding levels of performance in terms of quality, efficiency, equity and appropriateness [1, 2]. Indeed, this seems to hold true across the whole European Union since the EU Directive n. 24/2011 explicitly regulates EU citizens’ rights in terms of access to healthcare services across countries, as well as the required guarantees in terms of quality and safety of what is provided.

Indeed, a number of recommendations on how hospitals should improve their performance have emerged in time. The most obvious effort in this direction is perhaps given by the British Future Hospital Commission that highlighted the 11 core principles that should characterize hospitals in order to respond to their challenges and to develop a new model of care that delivers safe, high-quality care [3]. These include the main idea that patients’ experience should be valued as much as clinical effectiveness and that services should be tailored to meet their different types of needs.

Other recommendations which are specifically addressed to hospitals can, for example, be found in IESE’s Document “Hospital of the future”, and seem to suggest that hospitals should actively and pro-actively contribute to the design and implementation of organizational change. Among many other points, it is recommended that “*leading hospitals play an active role in helping public administration and society deal with the health care economics challenge, bringing vision and knowledge to the debate on the configuration of the future healthcare system. (…) Healthcare policy makers should consider including hospital clinical leaders when designing disease management strategies and plans, and consider including hospital managers when planning health care services*“ [4].

In other words hospitals are asked to play an active role in the achievement of their objectives (as well of the system’s ones) by providing a determinant support in the detection of the solutions to the pressures the system is facing. Nevertheless, although studies aimed at exploring specific aspects of change within hospitals, or across health care organizations, are numerous, scientific evaluations of the various approaches adopted, and a systematic effort to enhance knowledge and learning, are still somewhat limited [5]. Indeed, what seems to be missing is an effort to draw an exhaustive picture of the trends of change that the hospitals of developed economies are encountering, with reference both to the arising organizational and managerial solutions and to the tools to be used, and how, in order to implement them. For example, Lega and De Pietro [6] describe hospitals’ organizational change in terms of bridging (integration of different organizations), redesigning (integration among professionals) and engaging (directing behaviors towards the organization’s interests and goals), providing precious food for thought on the main directions of organizational change in the health care sector. Nevertheless, what still seems not to be present in the debate is a further reflection on making stock on what is known on how to *concretely* design and implement this change. Matters such as which organizational models and approaches should be adopted and *how* they should be effectively achieved, risk remaining partially unsolved due to the variety of points of view, features and aspects of change and interpretation of approaches that literature and experience provide.

Moreover, such ambiguity appears potentially harmful in the present context which seeks a new and more efficient balance between activities carried out within hospitals and those carried out by primary health care providers. On the one hand, the tremendous costs related to hospital care make it necessary for hospitals to focus on the acute moment of patients’ illnesses, relying more and more on primary settings for the other phases. On the other, this cannot be done in a “disjoint” way, meaning that hospitals cannot simply “entrust” patients to other settings after discharge, but must help to build-up a truly integrated system. Here, “*the hospital’s role will be not only coordinating but orchestrating services. In order to do that, hospital professionals will share clinical knowledge with other levels of care and providers in the network*” [4].

The purpose of this study is to make stock of what is known about how hospitals are changing in organizational terms, as well as of how such change may be implemented effectively through managerial tools. By doing so we provide insights on the existence of evidence on new organizational models’ effectiveness, and highlight gaps that research might still not have adequately dealt with.

After presenting the theoretical background and methodologies adopted in the study, we describe the main pillars of change in the Western world’s hospitals as well as exploring their features and drivers of implementation. The work is closed with the main considerations and conclusions emerging from such evidence.

## Background

Expenditure on health care is the largest single item of public spending in all the EU states, exerting strong pressure on public finances [7]. Member states have in place plans to reduce the rate of growth or even the absolute level of public expenditure, but these constraints come at a time when the population’s need for health care is growing quickly as a result of changing demography and changing paradigms for treatment. How these competing developments are to be managed constitutes one of the major challenges of EU member states.

Although the nature and trajectory of health policy in each country is intimately tied to each nation’s unique history, cultural values, political institutions and traditions, a number of convergent trends across countries can be discerned in relation to emergent approaches towards healthcare quality and patient safety [8]. In particular, there has been a shift from healthcare quality being viewed as a predominantly medical concern to the development of more organizational and managerial approaches towards promoting high-quality care [8]. Hence, the quality of care patients receive not only depends on the resources available and the cost and clinical effectiveness of treatments, but also on the internal organization and the integration of the structures within which they receive assistance. Clearly, integration and smoothness of pathways across settings require a high degree of coordination of different professions within single teams, but also of more teams delivering care along these care pathways, which typically involve both primary and secondary health care settings (e.g. community care and home care). Thus, integrated care requires understanding and matching skills, competencies and needs of different professions and teams, as well as removing barriers to the effective utilization of such skills through, for example, the enhancement of effective information exchange.

Indeed, organizational change has been widely explored in a large number of contexts, and quintessentially in healthcare organizations since if it is not well performed it can result in losses in the organization’s resources, negatively impact the quality of care patients receive and, ultimately, put at stake the organization’s actual survival [9]. Achieving change, which can be technological [10], of products or services offered, strategic and structural or, finally, cultural, seems to be one of the most difficult challenges management can face.

A possible solution to the risk of encountering decoupling phenomena could reside in the adoption of sociotechnical approaches in the design and management processes of the healthcare system. Indeed, this approach suggests that change should not be approached in terms of visions linked exclusively to technical means or to social ones. It suggests that doing so would provide only partial and non-credible results. Sociotechnical theory, therefore, is about joint optimization [11], that is, designing the social system and the technical one in tandem so that they work smoothly together. It is usually based on designing organizations in which the relationships between socio and technical elements lead to the emergence of productivity and wellbeing, rather than the unfortunately frequent case of new technologies failing to meet the expectations on their effectiveness.

In other words: “*Organizational objectives are best met not by the optimization of the technical system and the adaptation of a social system to it, but by the joint optimization of the technical and the social aspects, thus exploiting the adaptability and innovativeness of people in attaining goals instead of determining technically the manner in which these goals should be attained”* [12].

## Methods

Although the major trends in hospital organization are widely studied [6], relatively little effort seems to have been made in order to clearly codify these trends and provide a clear picture of the extent to which they have been implemented, and with what effects. More in particular, there still seems to be little evidence about which tools and enablers should be used in order to achieve such change.

**Fig. 1:**
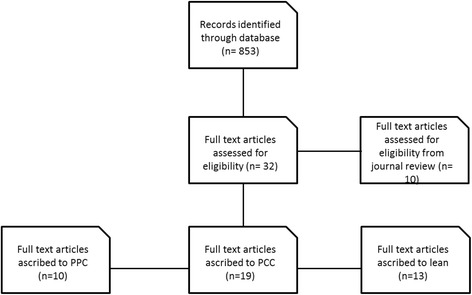
Literature flow chart

The methodology adopted in this work integrates a systematic literature review to a wider engaged research approach: the latter produced evidence through previous research and on-field experiences, such as consultation projects in healthcare organizations and participations to events and conferences. The systematic literature review was conducted with the “EBSCO” Information Services platform and “Web of Science”, with temporal lag 2000-2014. This temporal lag was chosen in order to select only contemporary evidence. We used the search string “(Hospital OR healthcare) AND (change management OR organizational model)” in article topics, in order to capture all the major trends of change within the healthcare sector. Only articles published in English or Italian were included as these are the languages fully mastered by the authors (Fig. 1). Further filters included web of science categories (health policy services) and research areas (health care sciences services) (Additional file 1).

Moreover, we conducted an analytical review of specific healthcare management journals, including BMC, Health Policy, Health Care Management Review, Journal of Healthcare Management. The abstracts of all the available articles were studied and pertinent articles were selected for the literature review. Duplicates were removed. All abstracts were reviewed by a single researcher. To assess relevance of the articles selected, the second researcher reviewed the abstracts in order to confirm their pertinence to the issues analyzed. There was complete agreement on the abstracts included in the study. This has been focused on the understanding of hospitals’ trends or “pillars” of organizational change, followed by an in-depth analysis for each of these of the enablers of change required to implement them concretely. Emerging themes have been detected through the extraction of definitions from each article. Where definitions were not clear and did not recall a recurrent theme, these were excluded from the analysis. The characteristics of the included studies are summarized in Table 1.

**Table 1:** Characteristics of the studies analyzed

Pillar of change	Author(s)	Year	Typology	Country
Progressive patient care model	Stacy	2011	Guidelines	USA
	O’Dea et al.	2003	Divulgative article	UK
	Quintero	2002	Guidelines	USA
	Berke, Ecklund	2002	Guidelines	USA
	De Pietro et al.	2011	Case-study	Italy
	Bohmer et al.	2010	Case study	France
	Lega, De Pietro	2005	Lit.rev./theoretical framework	Italy
	Orlandi et al.	2006	Case study	Italy
	Polimeni et al.	2003	Case study	Italy
	Villa et al.	2009	Case-study	Italy
Patient centered care approach	Halbesleben et al.	2010	comparative method	USA
	Johnson et al.	2012	Guidelines	USA
	Pearson	2001	Case study	USA
	Hernandez et al.	2013	Case study	USA
	Arbaje et al.	2010	Case study	USA
	Daaleman et al.	2014	Case study	USA
	Martínez-García et al.	2013	Case study	Spain
	Meijboom et al.	2011	Lit. Rev.	The Netherlands
	Vawdrey et al.	2011	Case study	USA
	Cruz-Correia et al.	2007	Lit. Rev.	Portugal
	Rothschild et al.	2005	Guidelines	USA
	Fieschi	2002	Lit. Rev.	France
	Daniel et al.	2013	Longitudinal	USA
	Rosemond et al.	2012	Case study	USA
	Tai-Seale et al.	2014	Cross sectional	USA
	Hearld, Alexander	2012	Cross sectional	USA
	Rathert et al.	2012	Lit. Rev.	USA
	Wu et al.	2012	Guidelines	Canada
	Villa et al.	2014	Guidelines	Italy
Lean approach	Waring, Bishop	2010	Ethnographic account	UK
	Radnor et al.	2012	Case study	UK
	Martens et al.	2014	Cross sectional	UK/Germany/France
	Yousri et al.	2011	Longitudinal	UK
	Cima et al.	2011	Longitudinal	USA
	Mazzocato et al.	2012	Case study	Sweden
	Vermeulen et al.	2014	Longitudinal	Canada
	Dickson et al.	2009	Longitudinal	USA
	Rees	2014	Case study	New Zealand
	McIntosh et al.	2014	Lit.Rev.	UK
	Skeldon et al.	2014	Longitudinal	Canada
	LaGanga	2011	Longitudinal	USA
	Burström	2014	Cross sectional	Sweden

## Results

### The main pillars of change

Our effort of trying to label the main trends in organizational and managerial approaches of hospitals resulted in the detection of three main pillars of change. We named these the progressive patient care model, the patient-centered approach and the lean approach.

The first has to do with the tendency to overcome organizational structures that are merely centered on specialty-driven clinical directorates, to switch to horizontal and transversal models. A number of different labels have emerged in order to name these new models. “Progressive patient care”, “comprehensive critical care”, “intensity of care models”, “care-focused organizations” [13] are all expressions that, although at times applicable to different contexts and possibly different in some aspects, ultimately lead back to one basic idea: pooling patients and organizing patient-flows around the acuity of patients’ conditions and not around the specialty they are concerned with. As Villa and colleagues put it, hospitals “*can no longer sustain functional self-referential designs, where resources are duplicated, economies of scale are underexploited, clinical integration and clinical governance is nonexistent, and autonomy (in using the specialty’s resources) prevails over accountability (on outcomes requiring the integration of different specialties in using fixed and shared resources, such as operating rooms, equipment, beds, and staff)*”. These old organizational models have caused too often inefficiencies in how staff is used, high rates of delay, cancellation of clinical procedures, and waste of resources resulting from poor communication among departments and disciplines [14–16].

Therefore, in a progressive patient care perspective, patient pooling is conducted on the basis of the degree of assistance the patient is in need of or on the likely duration of his/her stay in the hospital. This, in turn, implies that units should be merged into clinical directorates on the basis of requirements of care and potential economies of scales (and no longer, as mentioned, following specialty criteria). Moreover, it is easy to see how resources must now be shared among different specialties. These include staff, equipment, operating rooms and, ultimately, beds. Finally, a fundamental change introduced with the new model has to do with a new way of conceiving work, with assistance carried out by multi-disciplinary as well as multi-professional care teams, with nurses generally assuming new managerial and coordination responsibilities.

As mentioned, pooling of patients can take place on the basis of two criteria. The first has to do with their expected length-of-stay. Wards such as day-surgery/day-hospital, short-stay hospital, week hospital (for patients admitted on Mondays/Tuesdays and discharged within Saturdays), long stay-low care belong to this criterion. The second has to do with patients’ needs in terms of level of nursing assistance required or degree of dependency on medical equipment. For example, in Great Britain a new classification has been proposed based on four levels of patient dependency: level 0, patients receiving normal in-patient care with no special requirements; level 1, patients requiring additional monitoring and support above that which can be provided safely in an ordinary ward; level 2, patients requiring single organ system support excluding mechanical ventilation; and level 3, patients requiring multiple organ system support or mechanical ventilation [17].

The patient-centered approach is strictly connected to the new progressive patient care organizational model and consists in a “philosophy” that must drive the organization in order to make the new model work concretely. In other words, if with the progressive patient care model activities are organized within horizontal platforms and no longer in vertical clinical directorates, the patient-centered approach has to do with the way of “filling” these platforms with horizontal clinical pathways that determine patient flows. In particular, the focus must now be placed on the set of processes that determine such flows, and which include all core processes, referred to the clinical pathway of the patient, as well as support processes, which do not determine directly the objective of pursuing a status of health for the patient, but are still indeed interconnected with the primary clinical process [18]. These include processes such as, for example, pharmaceutical logistics, patient transportation or laboratory and imaging activities. It should be noticed that managing productive processes within the healthcare sector appears particularly challenging because of a number of factors [19–21] that characterize this sector’s processes in a unique way: in the first place “production” and “consumption” are of course simultaneous, with services which must be rendered in the precise moment they are requested. This means that in order to exploit at the maximum a hospital’s productive capacity, it is necessary to correctly manage both the supply of services, pursuing high levels of flexibility, as well as their demand, reducing as much as possible its variability. Secondly, production processes are extremely various and divergent, and often require an integration of very different professions and competencies. Moreover, these processes are also variable in terms of when and in what measure they may be needed. Finally, management must also consider the peculiarities of the professions involved in such processes, as for example the high decisional autonomy of physicians.

In other words, this approach aims at reshaping hospital care delivery processes around the needs of patients and away from the traditional physicians-centered view [22–24], in such a way that all (human, technical, etc.) resources merge into the pathway when needed by the patient, so that he/she must no longer “search” for what is needed. This must indeed be done across all the settings the patient is likely to cross (e.g. emergency departments, operating rooms, wards, intensive care units, post-acute care settings). A number of studies have explained how major problems of hospitals often depend on a poor management of patient flow, the most frequent probably being clinical mistakes, queues and delays, under- and over-capacity utilization, patient acceptance in inappropriate settings, variability of workload and stress for hospital staff [for example 6].

As a matter of fact, many authors suggest that hospitals should be viewed as complex systems made up of several internal sub-components that are tightly interdependent with each other [25, 26]. In order to pursue quality and efficiency, therefore, it is fundamental to globally address the overall cycle of care, from the patient’s first access to his discharge and follow-up, and not merely bits and pieces of the pathway, addressed in a disjoint way.

This brings us back to the progressive patient pooling approach, since a relevant aspect outlined by many studies is that patient flow problems are likely to occur when hospital resources (beds, operating room, human resources, etc.) are allocated in rigid ways and not regularly reallocated on the basis of actual patients’ needs [27].

Finally, the lean approach [28, 29] is again strictly connected to a patient-centered approach and, in turn, to a progressive patient care organizational model. If, as mentioned, the progressive patient care model can be thought of as an “organizational container” and the patient-centered approach as the set of pathways to “fill” such container, lean thinking should be conceived as the “kit of technical tools” necessary to effectively do so.

### Detecting existing evidence on the pillars of change

#### The progressive patient-care model

In reference to the progressive patient-care (PPC) approach, we found only partial coherence in terms of the meanings assigned to this specific label and, as mentioned, different labels to indicate similar concepts. In particular, a number of studies assign different names to the organizational model in which patients are no longer pooled on the basis of their prevalent pathology (i.e. in specialty- aggregated wards and clinical directorates), but instead on the basis of their expected length of stay within the hospital or of the degree of assistance they are in need of. The term “progressive care”, on the other hand, is sometimes referred to “progressive care units” (or step-down units) [30–33], intended as specific care units that lie in between intensive care settings on the one hand, and traditional clinical wards on the other.

Indeed, however, we have found a good enough coincidence in terminologies used across studies in order to be able to generalize our findings. A number of works described in detail how the progressive care model, intended as the progressive pooling system on the basis of the intensity of assistance required, has been implemented in specific contexts. For example De Pietro and colleagues describe how Tuscany (Italy) is a pioneer Region in the implementation of such an organizational model [34]. Indeed, the Region has imposed the model by law to all of its public health care organizations (in this region hospitals mainly belong to local health units and therefore the adoption concerns indiscriminately both settings), constituting therefore the main pressure for change. Although 3 levels of care are identified at the regional level (level 1, high intensity; level 2, medium intensity; level 3, low intensity which should be cared for outside hospitals’ walls), and in line with the evidence of most studies providing guide-lines and indications for implementation, great autonomy in defining *how* concretely the model should be developed and implemented is left to specific organizations. In turn, this could partly explain the wide range of ways of interpreting the model and of using enablers in order to achieve its desired effects.

A frequent decision is to re-organize hospitals in poles, i.e. aggregations of “old” clinical directorates in the intent of pursuing efficiency and quality of service. Such aggregation can take place on the basis of different criteria such as aggregation of “stronger” with “weaker” (in terms of revenue, for example) ones or complementarity of the activities carried out within departments [35].

Another frequent choice has to do with the decision of sharply separating elective patients’ from unscheduled ones’ pathways and, in particular, from emergency cases. This turns out to be useful in order not to excessively delay elective cases because of emergency “intrusions” and to better understand patient flow variability (across the day, the week and the year) using typical variability indicators [36].

Moreover, it is frequent to distinguish inpatient from outpatient (patients involved in ambulatory and day-activities) pathways, without the two categories ever “meeting” during their stay in the hospital [37]. Again, the objective here is to speed up pathways, reducing queues and bottlenecks.

As a matter of fact, studies pose a great emphasis on the emergency department, often considered the hub of change. Indeed, this seems to be the “engine” and the driving force of new organizational models. Understandably, the sustainability of horizontal, swift, and patient-oriented processes have a lot to do with a correct triage activity and the recognition of the appropriate pathways to be followed. In particular, with the new progressive care model, the emergency department becomes responsible of correctly assigning patients to the “different levels of care” within the hospital, and is therefore the main responsible of possible admissions in inappropriate settings.

To overcome possible mismatches between demand and supply of beds, hospitals can often count on a number of “pool beds” that are set aside to accommodate patients that, for different reasons, are outside the different pipelines (for example, patients that were admitted to a week-surgery but, for some reason, need to stay in the hospital more than 5 days) [13].

A final frequent organizational innovation has to do with the decision to centralize as many functions and services as possible. Usually these include the sterilization units and the operating rooms, as well as all the activities related to pre-admission testing [13].

In reference to the most adopted drivers to implement change, we found examples of Local Institutional Authorities explicitly requiring procedures designed on the new settings in order to exert clinical activities on behalf of the public system [34]. In concrete, this correspondence seems to consist in the adoption of Integrated Planning, Budgeting and Control Systems such as, for example, the Balanced Scorecard (or similar tools) which, if correctly implemented, require a fair negotiation of goals and key (critical) performance indicators with many hierarchical levels [6]. In concrete, however, managerial accountability tools (such as, for example, budgets that are still assigned to clinical wards) are usually not aligned to the new organizational model [34].

Evidence was found in terms of the emergence of new professional roles and a general reassignment of responsibilities among individuals and professions. Indeed, the trend seems to converge towards new multi-disciplinary roles with nurses covering more and more managerial and coordination tasks. For example in Tuscany old specialty-aggregated clinical directorates are replaced by “functional areas”, which are directly in charge of beds, staff, technologies and resources in general. Concretely, these resources are managed by the areas’ “nurse coordinators”, who are now responsible of their appropriate and efficient use. In general, nurses (or other health professionals) are often asked to cover a range of new managerial roles such as [13, 32, 34]:

- Admissions coordinator: in charge of the pre-recovery process and of the admission procedures;
- Hospital rounds coordinator: in charge of the coordination of the visits to the ward by the different specialties;
- Supply coordinator: in charge of managing the logistic flows of goods (pharmaceuticals, medical devices, and other materials) to the different wards;
- Operating room coordinator: in charge of assigning and controlling the use of the operating theatres;
- Bed manager/facilitator: in charge of establishing efficient patient placements in the different inpatient settings;
- Clinical directorate coordinator: in charge of the general coordination of activities within clinical directorates;
- Quality responsible: in charge of coordinating and controlling quality management activities;
- Training and Education coordinator: in charge of coordinating and evaluating nurses’ formation;
- Nursing tutor/case manager: in charge of “following” a patient through all of his/her pathway and of correctly coordinating all the activities and professionals involved in it, as well as providing constant guidance and support to the patient himself and to his/her family.

It is not only through the achievement of new managerial roles that nurses assist to a deep change in their profile. Actually, also those nurses (who are the majority) who keep on providing clinical assistance face drastic changes in their jobs as a consequence of the new organizational model. Indeed, they must achieve the ability of working in new multidisciplinary settings in which their competencies and clinical knowledge is likely not to cover the vast range of pathologies and case mixes treated in the new horizontal platforms. Moreover, nurses who carry out triage activities in Emergency Departments assume a great responsibility in correctly allocating patients in the various “levels” innovative hospitals now hold on. Also, some new coordination roles cannot be carried out without an ample knowledge on various clinical fields. For example, bed managers collaborate with the medical staff to assess patients’ needs and appropriate placement of individual patients. Therefore, they are required to use operational and clinical judgment on a daily basis to prioritize bed assignment and reassignment.

Organizations have usually met this challenge by organizing training activities for specific settings (not rarely required by the professionals themselves) and efforts have been made in order to allocate nurses who had been working in either medical or surgical wards in coherent settings [34]. Moreover, nurses with a recognized experience on specific specialties or pathologies are usually assigned to specific settings, in order to guarantee within them the presence of some highly specialized figures.

Physicians also seem to face deep changes in their professions. Again, as is the case for nurses, they are sometimes called to cover new roles such as, in the first place, heads of new organizational settings (i.e. High Care, Week Surgery, Week Hospital, Urgency Medicine and Post-Acute Care) [13]. Moreover, medical tutors/medical case managers become responsible of the whole medical pathway the patient follows, as well as being the reference point he/she as well as the family can rely on at any stage of the pathway [32].

All these evolutions, together with the always critical necessity to manage individual skills, push for a gradual adoption of a competency-based model for HRM (through a competency-based model it is possible to manage all the competences discussed above, ranging from the more technical/clinical ones to the managerial and organizational ones). Moreover, hospitals and their sub-units (horizontal settings, clinical directorates, specialties, etc.) need to design and develop career paths and, therefore, appraisal systems in order to manage promotions along the professional line or the managerial line. Finally, it is felt that the evaluations of individual and team performances cannot be limited to sporadic events, nor can they be carried out by a single role. On the contrary, it is held to be important to enhance a multi-source and 360° feedback system, with evaluations expressed by many actors such as, for example, the chief of department, the chief of nurses, the manager of specialty, ward managers, peers, collaborators, patients. It is believed that this approach is better accepted by professionals and more likely to orient behaviors towards the organizational goals [6].

Finally, implementations of the progressive care model highly rely on innovative ICT tools which enable a swift and accurate communication among actors. In particular, the most frequent tools adopted are integrated electronic health records, to be jointly used and updated by physicians and nurses [34].

Evidence suggests that the new organizational model has in many cases led to good results. There exist numerous examples of improved efficiency indicators such as reduced waiting times, reduced stockpiles, reduced bureaucratic procedures and duplicated information [34, 35, 37], as well as reductions in average hospital lengths of stay, increased bed occupancy rates, increased hospital case-mix complexity, reduction in turn-over ratios, increase in patient inflows, that is, patients coming from different catchment areas [13] although at times this occurs at the cost of putting more strain on employees [35].

Moreover, results report an increased patient satisfaction [13], especially in reference to the identification of a medical and/or nursing tutor (yet not in reference to the general stay in the hospital) [34]. Patients are reported to no longer being “parked” in areas where they cannot receive appropriate care. Indeed, a multi-disciplinary approach seems to be strongly encouraged and collaboration between the medical and surgical staff seems to improve [13, 32]. The logics of process management is further enforced and thus promotes the development of care maps and clinical pathways [13]. However, there is a lack of evidence in terms of improved clinical outcomes.

Other reported unsolved problems have to do with: the definition of a more clear repartition of medical and legal responsibilities among medical tutor and other physicians, as well as between physicians that manage different platforms; the optimization of bed capacity exploitation, especially in reference to an effective and efficient allocation of patients coming from the ED; an effective allocation of nurses to different settings on the basis of the concrete intensity of assistance required by their patients; the implementation in EDs of trustworthy assessment tools which enable a correct evaluation of the degree of intensity of care required.

Open issues also have to do with the assessment of the desirability of medical day- or week- hospitals, given the general difficulty of predicting the expected length of stay of medical patients, and with the capability of actively involving professionals and of overcoming cultural barriers, especially on the physicians’ side [13, 37].

#### The Patient-centered approach

Although broadly studied for decades, a clear definition of patient centered care (PCC), as well as an understanding of how specific PCC processes relate to patient outcomes is lacking [38].

Yet we were able to find a relatively numerous set of documents in which the definition of patient-centeredness is (at least partially) coherent with the meaning we assigned to it. Indeed, most articles analyzed do intend PCC as the tendency to organize activities along and around the patient’s pathway, as opposed to an approach in which patients must go and seek the services they need in specific physical and organizational locations. However, in some cases, and mostly in reference to articles from the USA, PCC assumes a broader meaning and also includes the idea of enhancement of positive relationships between care providers and patients by promoting daily routines that are tailored to their life experiences, abilities and preferences [39].

Moreover, the topic seems to be poorly explored within hospitals’ walls, and somewhat more studied within primary health care settings. In particular, and again especially in the USA, scholars’ attention is often focused on Patient Centered Medical Homes, intended as medical homes that ideally tailor and individualize health care services to patient needs by increasing access and managing all aspects of care [40].

An exception has to do with the analysis of general process improvements within EDs, that constitute one of the most studied settings within hospitals. For example, a study suggests how important it is, in order to improve processes, to starkly invest in determining the “voice of the customer” – intended as patients' and staff’s perceptions- by using internal survey tools or external services [41].

The importance of designing and managing smooth processes is confirmed by studies analyzing the effects of suboptimal ones. For example, a study explains that health care professionals encountering barriers within processes can choose to either engage in workarounds to get past the block, or potentially repeat work (rework). Both solutions are likely to cause waste and lead to safety concerns. In particular, issues related to information exchange tend to lead to rework, internal supply chain issues are more likely to lead to workarounds [42]. Moreover, the causes of distorted processes and pathways often have to do with inadequate allocation of capacity as well as a lack of coordination between different pipelines and production units [36]. Policy, it is suggested, should stimulate the provision of more coordinated services, for example, through integral cost prices for separate diseases (“case-mixed accounting”) [43].

Anyhow, the most analyzed aspect of PCC is related to continuity of care among different settings. As a matter of fact, patients commonly experience a complete new set of caregivers as they progress from acute to subacute care settings. Managing continuity of care, as well as guaranteeing accountability for overall outcomes becomes challenging, and patients often feel abandoned by their primary caretakers. The most frequent response to this issue is given by the assignment of care coordinators or of rehabilitation liaison nurses, in charge of following patients from the acute to the sub-acute setting [43, 44]. In other cases, nurse practitioner teams assess patients, co-manage syndromes, provide staff education, encourage patient self-management, communicate with primary care providers, and follow up with patients soon after discharge [45].

The main enablers to implement PCC appear to include an effective leadership and management communication, with the necessary technical and professional expertise and creative/soft skills; a strong internal and external motivation to change (favorable perceptions from direct care providers about the priority of the innovation to the organization); a clear and internally consistent organizational mission; an aligned organizational strategy; a robust organizational capability; and a continuous feedback and organizational learning [39, 46].

ICT tools should ideally be able to improve workflow through the prioritization of information and detection of individuals’ contextual situations, promote stronger inter-professional relationships with adequate exchange of information, enable interoperability and scalability between and within institutions, function across different platforms [47]. This is particularly relevant for multi-morbidity patients’ care because there is a large number of health professionals in charge of patient care, and this requires to obtain clinical consensus in their decisions [48].

There exist a few pioneer experiences in the implementation of innovative ICT tools. For example, a study reports the experience of the implementation of a Shared Care Platform within a hospital and two primary care centers to provide support in the continuity of care for multi-morbidity patients. This platform includes a social network component (the Clinical Wall) which contains a record where health professionals are able to debate and define shared decisions. Preliminary results suggest that such type of tool can indeed enhance communication effectively, having during its pilot implementation phase favored decisions about coordination for appointment changing, patient conditions, diagnosis tests, and prescription changes and renewal [48].

ICT tools, anyhow, still appear to be rudimental if compared to their potential, as is testified by studies that explain how not only the information they deliver (through, for example, electronic health records) are often not exhaustive [49], but also how they often fail to even identify numerous individuals involved in patients’ care, suggesting that electronic health records may not provide adequate tools for care team designation [50].

In particular, it is suggested that “*the failure to view the hospital as a system has contributed to the practice of inefficient and ineffective clinical documentation. Rethinking IT in support of clinical documentation from a system-oriented perspective may help improve patient care and provider communication*” [49]. The suggestion is to design systems in which “*the clinician first enters the patient’s relevant problems and subsequently performs other actions in the explicit context of the single most relevant problem to which they relate. The problem thus drives the care and (…) an interdisciplinary problem-oriented view would keep all providers focused on the whole patient and defragment clinical care*” [49].

The most relevant challenge communications systems seem to face has to do with a switch from hospital information systems to health care information systems, in coherence with collective decision-taking processes [51]. The integration of Information Systems seems indeed to be essential to support shared care and to provide consistent care to individuals – i.e. PCC [52].

Although *liason* figures seem to be appreciated and effective in enhancing communication among professionals (in a study a majority of physicians (75%) and support staff (82%) interviewed reported interactions with a care manager [40]), in general evidence about the effects of PCC on clinical outcomes seem to be very limited. Some studies found significant relationships between specific elements of PC and outcomes [38, 53], others between a patient-centered approach in general and a reduced ED utilization, due to an improved care coordination and reduced delays in care [54]. Another study suggests that *liason* activities are associated with slightly higher, though not statistically significantly so, quality care transitions and greater patient satisfaction with inpatient care [45]. In general, however, evidence seems to suggest no significant and universally recognizable relationship [38]. Improved continuity of care, anyhow, as well as transdisciplinary teams’ shared resources, have been found to increase patients’ satisfaction through a gained sense of support throughout the continuum of care [44].

All in all, tools to identify the concrete degree of patient centeredness implementation are rare, although a few attempts have indeed been made, such as, for example, the Patient-Centered Medical Home Assessment tool, aimed at stimulating and monitoring progress among primary care practices interested in transforming to patient-centered medical homes [55]. This sort of experience, however, was not detected in hospital settings.

#### The lean approach

Vast research has been dedicated to the implementation of lean methodologies within healthcare systems, often suggesting their benefits in resource utilization and patient care [56–58]. There seems to be agreement on the factors that determine the extended array of improvements reported. In particular, factors such as standardized work and reduced ambiguity, new connections between people who depend one form another, enhanced uninterrupted flows through processes, and empowered staff to investigate problems and to develop countermeasures using a “scientific method”, seem to be the keys to success [59, 60]. Moreover, process mapping, leadership support, staff engagement, and sharing performance metrics are felt to be keys to enhancing efficiency [61].

What seems to make the difference is that management assumes a subordinate role when it comes to solving flow issues, adopting a “bottom-up approach”, by allowing the frontline staff to identify problems and come up with appropriate solutions. The risk, otherwise, is to face reluctant staff, who feels forced to institute top-down process improvements, with a perception of being monitored [59, 60].

Once again, experiences are often referred to lean projects within EDs. In a Swedish pediatric Accident and emergency department, for example, lean-inspired changes to employee roles, staffing and scheduling, communication and coordination, expertise, workspace layout, and problem solving led to improvements in waiting and lead times (in the order of 19-24%) which were sustained in the 2 years following change [60]. In two different EDs a Quality Improvement project that included lean principles improved the self-estimated patient safety culture, mainly due to team-work and communication openness [62]. In a further ED in which lean techniques were implemented through a six-step process of Lean education, ED observation, patient flow analysis, process redesign, new process testing, and full implementation, patient visits increased by 9.23% and, despite this, length of stay decreased slightly. Moreover, patient satisfaction increased significantly without raising the inflation adjusted cost per patient [59].

Evidence about the application of Lean and Six Sigma methodologies within operating rooms (ORs) and across surgical suites have also been reported. A study, for example, reports substantial improvements in on-time starts and reduction in number of cases past 5 PM, as well as substantial gains in non-operative time, staff overtime, and ORs saved. These changes, in turn, resulted in substantial increases in margin/OR/day [61].

In an outpatient setting, a 3-day value stream analysis and a 5-day rapid improvement event were able to shorten the patient cycle time and the time to initial assessment [63]. In another outpatient service a lean process improvement project brought to a 27% increase in service capacity to intake new patients and a 12% reduction in the no-show rate [64].

Some successful experiences are also referred to transversal patients’ pathways across different settings (within the hospital and across structures). In five European hospitals, for example, there was a 59% reduction in the average time to diagnosis and a 75% increase in diagnostic yield in response to the implementation of a structured Lean Six Sigma based methodology to pathways for syncope management. Moreover, a marked reduction in repetitions of diagnostic tests and an improved prioritization of indicated tests were also recorded [65].

A statistically significant reduction of 5% and 9.3% was noted in the 30- day and overall mortality, respectively, after implementing ‘Lean thinking’ in the management of hip fracture patients in a hospital trust. Further improvements were also reported in door-to-theatre time, use of trauma beds and early discharge from hospitals [66].

Despite numerous examples of successful implementations of lean techniques, literature does not fully agree on their degree of success within the healthcare sector, often finding inconsistent the evidence about their contribution to higher organizational performance. For example, a recent study suggests that “*a progressive managerial philosophy has a stronger impact on healthcare performance than the adoption of practices from any particular managerial approach*” (including lean). It argues that the most successful adaptations occur when employees manage the steps that produce value as a whole, rather than in bits or silos, with the organizational implications that productivity measurement should be carried out at the system level rather than by unit. This approach however is rare and there are no lean implementations across an entire hospital [67]. Therefore it is suggested to interpret evidence with extreme caution [68].

As a matter of fact, a further study that analyzed the effects of an ED process improvement program based on lean methods found that although the program reduced ED waiting times, it appeared that its benefits were diminished or disappeared when compared to control sites that had not implemented the program but had been exposed to system-wide initiatives such as public reporting and pay for performance. Again, the suggestion is to further evaluate the effectiveness of lean methods before spreading out its implementation [69].

In particular, despite a relevant number of successful implementations of lean projects is reported, lean is often felt to be “*a constellation of disjointed and poorly connected activities (…) which tends to involve the application of a narrow range of specific tools or techniques (…). Leaders tend to understand Lean as a collection of stand-alone, operational tools, rather than as a broader system-wide improvement philosophy*” [70]. Approaches such as ‘kaizen blitz’ or ‘rapid improvement events’ seem to have dominated the healthcare sector’s scenario, leaving sporadic experiences in terms of fully re-designing, in a holistic perspective, the whole set of processes that constitute pathways across organizations [70, 71]. In other words, “*while a project management implementation methodology may make many gains initially, sustaining the gains relies on the project’s integration and identifying what is needed for any changes to become routinized*” [72].

Indeed, research seems to suggest that the implementation of lean within the sector is often difficult and risks providing disappointing results if not sustained in time. In particular, organizational readiness, an adequate organizational culture, effective leadership (given the difficulty of effectively enrolling staff in the change agenda) and the availability of adequate resources and communication strategies appear to be fundamental for its success [57, 72, 73]. Moreover, lean must overcome important lines of resistance, which often see clinicians apprehensive about the motives and legitimacy of change as well as concerned about the validity of theories suggesting benefits for patients due to changed working practices [73]. Evidence suggests that lean is often poorly understood by who has to implement it and can be felt as a threat to personal freedom and autonomy, with the risk of being perceived as a pressing form of control on one’s work [60]. The “bottom-up approach” mentioned above, therefore, seems not to be fully implemented in concrete.

Further factors that may have impeded a greater improvement seem to include a mismatch between job tasks, and discomfort with inter-professional collaboration [60], suggesting that multidisciplinary formation should be improved, in order for professionals to feel more comfortable in actively collaborating with other professions.

Table 2 presents a summary of the main evidence reported by scientific literature in reference to the progressive patient care organizational model, the patient-centered approach and the lean approach, as well as the drivers of change necessary to effectively implement them within hospitals.

**Table 2:** Main evidence reported by scientific literature

	General evidence	Managerial Accounting tools	HRM tools	ICT tools	Outcomes	Open issues
Progressive patient care model	In some contexts implementation by law (e.g. Tuscany) Great autonomy of organizations on how to implement model Forms of implementation: poles; separation elective/unscheduled/emergency patients; distinct inpatient/outpatient pathways; emphasis on ED; pool beds; centralization of functions	Integrated Planning, Budgeting and Control systems (e.g. BSC) In concrete, however, MA tools not aligned to model (e.g. budgets still assigned to clinical wards)	New professional roles and a general reassignment of responsibilities (nurses and physicians) Need for: Competency based model; Separate professional/managerial career paths; Multi-source and 360° feedback system	Integrated electronic health records, to be jointly used and updated by physicians and nurses	Improved efficiency indicators Increased patient satisfaction (medical and/or nursing tutor) More coordination between medical and surgical staff Better implementation of clinical pathways Lack of evidence in terms of improved clinical outcomes	Effective allocation of nurses to different settings Correct triage activity in ED and efficient allocations of patients Desirability of medical day- or week- hospitals Involving professionals and of overcoming cultural barriers Definition of clear repartition of responsibilities among professionals
Patient centered approach	The most analyzed aspect of PC is related to continuity of care among different settings (poorly explored within hospitals) An exception: analysis of general process improvements within EDs		New professional roles in hospitals (e.g. liason nurse)	ICT tools should ideally: prioritize information and detect individuals’ contextual situations, promote stronger inter-professional relationships with adequate exchange of information, enable interoperability and scalability between and within institutions, function across different platforms. Few pioneer experiences (e.g. Shared care platform) ICT tools still rudimental if compared to their potential	Significant relationships between specific elements of PC and outcomes, or between PC approach in general and a reduced ED utilization. Liason activities are associated with slightly higher (not significant), quality care transitions. Greater patient satisfaction Improved communication among professionals General evidence about the effects of PCC on clinical outcomes very limited	Poor attention of literature to PC within hospitals Necessary switch from hospital information systems to health care information systems Non exhaustive information Individuals involved are not traced Lack of tools to clearly assess PC
Lean approach	Application of various features of lean such as new employee roles, staffing and scheduling, communication and coordination, workspace layout, process design etc. Application of lean tools within settings (EDs, ORs, outpatient settings) Only few examples of lean applied to pathways		Staff empowerment and “bottom-up approach”, by allowing the frontline staff to identify problems and come up with appropriate solutions		Many examples of improvement in efficiency indicators Fewer examples of improvement in clinical outcomes A number of studies find inconsistent the evidence about lean’s contribution to higher organizational performance	Lean is often felt to be “a constellation of disjointed and poorly connected activities” Lack of “system-wide” improvement philosophy The “bottom-up approach” is not fully implemented in concrete and barriers to implementation persist Need of more formation for inter-professional collaboration

## Conclusions

This work aimed at taking stock of what is known and reported about the major organizational and cultural trends that are characterizing hospitals in the Western world. It is not frequent to find efforts in sketching them globally, being research usually attentive to highlight and analyze specific and isolated aspects, initiatives, projects or settings.

The effort of labeling the main features of change resulted in the detection of three main pillars of change. The first has to do with a new way of pooling patients within hospitals’ walls, which we named the progressive patient care model. The second is the patient-centered approach, characterized by the tendency of organizing activities “around” the patient. The third is the lean approach, which has to do with the idea of carrying out activities smoothly, reducing bottlenecks and wastes.

Moreover, evidence seems to converge around three “families” of tools that should support the implementation of such change. These are Information Communication Technology (ICT) tools, Managerial Accounting (MA) tools and Human Resource Management (HRM) tools. Interestingly, these seem to cover both the technical and social dimensions of change pursued by the sociotechnical approach mentioned above.

Specifically, the technical one includes ICT tools which, it is suggested, not only should be adequate to support the information flows requested by organizational model, but should also be adequately used by the right people, avoiding mistakes, delays and unnecessary costs.

Instead, the social drivers of change are related to Managerial Accounting (MA) tools and to Human Resource Management (HRM) processes and tools, in coherence with the belief that these dimensions are crucial in achieving successful implementations of an organizational model [74]. It is worth to be noted that a structured and effective managerial accounting system should starkly support a clear repartition of responsibilities among settings and among professionals. An (also only partial) inability to gather and decode effectively the information necessary to judge performance may have dramatic consequences in terms of “losing sight” of strategy and of achieving organizational goals. Furthermore, HRM is made up of a number of conceptually different and sequential phases which must however all be effective, being the risk that if there exists also only one weak ring in the chain, the whole process of change may be damaged. These phases include people’s selection, allocation, evaluation, reward, training, retain and lay-off. Indeed, vast evidence exists on how poor human resource management may result in a *de facto* impediment to the successful implementation of even the most promising organizational model [75, 76].

**Fig. 2:**
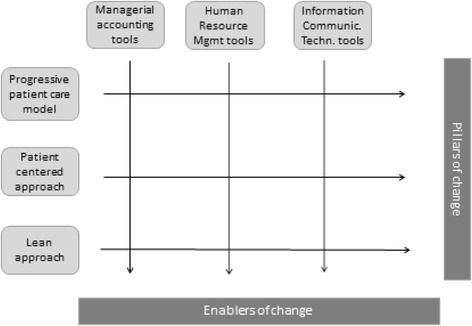
Health care organizations’ trends of change and the tools to achieve it: a conceptual framework

In Fig. 2 we present the general conceptual framework emerging from this research.

The progressive patient care model can still rest on very poor evidence both in reference to how to implement it and on the results it is able to achieve, especially in terms of clinical outcomes. Although integrated planning, budgeting and control systems are recommended, usually managerial tools are fragmented and obsolete and fail in giving an integrated orientation to organizations’ managerial accounting activities.

The emergent professional roles the model implies have been scrupulously traced and described. However, a lot less effort has been invested in producing guidance on how to select, train, evaluate and reward them. In particular, how to concretely manage career pathways remains an open issue. Exploring the patient-centered approach seems to confirm the trend. Although it is clear what new roles are needed (for all, *liason* nurses and other figures that can guarantee continuity of care among intra- and inter-hospital settings, as well as in local settings), *how* these people should be managed within the organization is still ambiguous.

A strong need of integration of ICT tools among settings and organizations, in a “system-wide” perspective, is clearly detected. A few examples of innovative solutions have been reported and, at times, implemented in specific contexts. These, however, are sporadic and the general situation seems to be characterized by multiple and often ineffective communication tools, that are unable to guarantee a smooth and exhaustive communication flow among professionals who interact with a patient in different moments and in different places. We found no evidence of significant investments at a local or regional level in reference to integrated ICT systems.

Again, the patient-centered approach seems to suggest improvements in efficiency indicators, but very limited evidence has been detected in reference to its effects on clinical outcomes.

In reference to “lean-thinking”, works studying its implementation and effects are abundant. However, a few remarks need to be made. In the first place, evidence produced seems to concern a lot more gains in efficiency matters than, again, in clinical ones. Moreover, the evidence that has been produced is referred to the implementation of specific and “one-shot” projects, or to the effects in specific settings (EDs, ORs, etc.) within a hospital or, at most, the effects on specific clinical pathways. As suggested, what seems to be missing is an effort to study lean at a hospital- wide and system-wide level. In particular, efforts to judge its global implementation are difficult to find. As mentioned frequently, this risks to undermine any form of generalizability of results observed in punctual contexts. Anyhow, studies aimed at exploring how to enhance the drivers of change to implement lean thinking are nearly absent.

Generally, more clarity is needed on how to redesign managerial accounting tools that are able to fit in the new hospital the model and the approaches imply. Although coherence between managerial accounting tools and new internal settings is hoped for, we found poor examples of integrated management-control and performance-management systems within hospitals. Indeed, integrated tools such as the Balanced Scorecard would seem to enhance a “global vision” of organizations, reducing the highlighted risk of incurring into policies that take into account only “bits and pieces” of the system, failing therefore to consider their effects extensively. Moreover, such a tool would imply an active participation of many hierarchical levels in the definition of an organization’s objectives, pursuing the very longed for involvement of human resources within the implementation of new organizational approaches.

Furthermore, the emergence of new professional profiles is clear, but little is known on how to correctly manage them, just as poor is the experience in terms of effective integrated communication systems to support such new roles in their activities.

Finally, this study confirms that efforts in literature are concentrated in analyzing circumscribed experiences in the implementation of new models and approaches, failing therefore to extend the analysis at the organizational and inter-organizational level in order to legitimately draw consequences to be generalized. There seem to be a number of “gaps” in what is known about how to exploit drivers of change and their effects, suggesting that the research approach privileged till now fails in providing a clear guidance to policy makers and to organizations’ management on *how* to concretely and effectively implement new organizational models.

This preliminary evidence, therefore, suggests that studies aimed at systemizing, comparing and also evaluating the “global” effectiveness of new organizational models in hospitals should be highly encouraged. This seems to be the only way to produce knowledge on how to concretely achieve the very longed for organizational smoothness required by the system. In turn, producing scientific evidence in this direction would probably be the enabling key for hospitals to effectively cover the active and pro-active role assigned to them in not only implementing but also designing the health care system’s future scenario.

## Additional file

Additional file 1: Search strategy and filters. (DOCX 12 kb)

## Abbreviations

ED: Emergency department
HRM: Human resource management
ICT: Information communication technology
MA: Managerial accounting
PCC: Patient centered care
PPC: Progressive patient-care

## References

[1] Freedman DB (2002). Clinical governance—bridging management and clinical approaches to quality in the UK. Clin Chim Acta.

[2] Scally G, Donaldson LJ (1998). Clinical governance and the drive for quality improvement in the new NHS in England. Br Med J.

[3] Future hospital commission. “Future hospital: Caring for medical patients” 2013.

[4] Ribera J, Antoja G, Rosenmoeller M., Borras P. Hospital of the future: a new role for leading hospitals in Europe. 2016.

[5] Groene O, Klazinga N, Wagner C, Arah OA, Thompson A, Bruneau C, Suñol R (2010). Investigating organizational quality improvement systems, patient empowerment, organizational culture, professional involvement and the quality of care in European hospitals: the ‘Deepening our Understanding of Quality Improvement in Europe (DUQuE)’ project. BMC Health Serv Res.

[6] Lega F, DE Pietro C (2005). Converging patterns in hospital organization: beyond the professional bureaucracy. Health Policy.

[7] OECD, Health at a Glance, 2009.

[8] Braithwaite J, Matsuyama Y, Manninon R, Johnson J. Healthcare Reform, Quality and Safety. Perspectives, Participants, Partnerships and Prospects in 30 Countries. Ashgate. 2015.

[9] Jacobs S, Weiner B, Reeve B, Hofmann D, Christian M, Weinberger M (2015). Determining the predictors of innovation implementation in healthcare: a quantitative analysis of implementation effectiveness. BMC Health Serv Res.

[10] Armenakis AA, Bernerth JB, Pitts J, Walker HJ (2007). Organizational change recipients’ belief scale: development of an assessment instrument. J Appl Behav Sci.

[11] Petrakaki D, Cornford T, Klecun E. Sociotechnical Changing in Healthcare. In Information Technology in Health Care: Socio-Technical Approaches. C Nohr and J Aarts. Amsterdam: IOS Press: 2010.

[12] Cherns A (1976). The principles of sociotechnical design. Human Relations.

[13] Villa S, Barbieri M, Lega F (2009). Restructuring patient flow logistics around patient care needs: implications and practicalities from three critical cases. Health Care Manag Sci.

[14] Mintzberg H (1997). Toward healthier hospitals. Health Care Manage Rev.

[15] Norrish B, Rundall T (2001). Hospital restructuring and the work of registered nurses. Milbank Q.

[16] Lega F (2007). Organisational design for health integrated delivery systems: theory and practice. Health Policy.

[17] O’Dea J, Pepperman M, Bion J (2003). Comprehensive critical care: a national strategic framework in all but name. Intensive Care Med.

[18] Villa S (2012). L’Operations management a support del Sistema di operazioni aziendali. Modelli di analisi e soluzioni progettuali per il settore sanitario. CEDAM..

[19] Borgonovi E, Zangrandi A (1988). L’ospedale.

[20] Vissers J, Beech R (2005). Health operations management.

[21] Boscolo P, Giusepi I, Marsilio M, Villa S, Anessi Pessina E, Cantù E (2011). Innovazione e performance nella gestione della supply chain in sanità: esempi nazionali ed internazionali a confronto. L’aziendalizzazione della sanità in Italia.

[22] Buchan J, Hancock C, Rafferty A (1997). Health sector reform and trends in the United Kingdom hospital workforce. Med Care.

[23] Coulson-Thomas C (1997). Re-engineering hospital and healthcare processes. Health Estate J.

[24] Plsek P (1997). Systematic design of healthcare processes. Qual Health Care.

[25] Litvak N, van Rijsbergen M, Boucherie R, van Houdenhoven M (2008). Man-aging the overflow of intensive care patients. Eur J Oper Res.

[26] Zonderland M, Boucherie R, Litvak N, Vleggeert-Lankamp C (2010). Planning and scheduling of semi-urgent surgeries. Health Care Manag Sci.

[27] Walley P, Steyn R (2006). Managing variation in demand: lessons fromthe UK National Health Service. J Healthc Manag.

[28] Fillingham D, Lean Healthcare: Improving the patient's experience. Chichester: Kingsham Press; 2008.

[29] Nicosia F (2010). L’ospedale snello.

[30] Quintero J (2002). Achieve cost benefits with innovative care management. New literature suggests progressive care units decrease costs, generate revenue, and help health care leaders better manage utilization of scarce Icu beds. Nurs Manag.

[31] Berke W, Ecklund M (2002). Progressive care units continue to grow in number as the patient acuity gap between critical care and medical/surgical care narrows. Nurs Manage..

[32] Orlandi W, Duca E, Pioppo M (2006). L’ospedale per aree di intensità di cura omogenee e di assistenza multispecialistica: l’esperienza dell’Azienda usl n. 3 dell’Umbria. Organizzazione Sanitaria.

[33] Stacy K (2011). Progressive care units: different but the same. Crit Care Nurse.

[34] de Pietro C, Benvenuti C, Sartirana M. Gli ospedali per intensità di cura in Toscana: un’esperienza in corso. L’aziendalizzazione della sanità in Italia. Rapporto Oasi. 2011;1:413-34.

[35] BOHMER R., BEYERSDORFER D., HARROW S., 2010. Hopital de Pointoise. Harvard Business School Review, 9-610-100

[36] Villa S, Prenestini A, Giusepi I (2014). A framework to analyze hospital-wide patient flow logistics: evidence from an Italian comparative study. Health Policy.

[37] Polimeni J, Lega F, de Lucis S, Fraccaro S, Gherardi F, Sosio F (2003). Nuove prospettive nell’organizzazione dell’ospedale generale di comunità: il caso dell’Ospedale di Pontedera. Organizzazione Sanitaria.

[38] Rathert C, Wyrwich M, Boren S (2012). Patient-centered care and outcomes: a systematic review of the literature. Med Care Res Rev.

[39] Rosemond C, Hanson L, Ennett S, Schenck A, Weiner B (2012). Implementing person-centered care in nursing homes. Health Care Manage Rev.

[40] Daaleman T, Hay S, Prentice A, Gwynne M (2014). Embedding care management in the medical home: a case study. J Prim Care Community Health.

[41] Johnson M, Capasso V (2012). Improving patient flow through the emergency department. J Healthc Manag.

[42] Halbesleben J, Savage G, Wakefield D, Wakefield B (2010). Rework and workarounds in nurse medication administration process: implications for work processes and patient safety. Health Care Manage Rev.

[43] Meijboom B, Schmidt-Bakx S, Westert G (2011). Supply chain management practices for improving patient-oriented care. Supply Chain Manag An Int J.

[44] Pearson J (2001). Extending a rehabilitation pathway to include multiple providers: outcomes and pitfalls. Rehabil Nurs.

[45] Arbaje A, Maron D, Yu Q, Wendel V, Tanner E, Boult C, Eubank K, Durso S (2010). The geriatric floating interdisciplinary transition team. J Am Geriatr Soc.

[46] Hernandez S, Conrad D, Marcus-Smith M, Reed P, Watts C. Patient-centered innovation in health care organizations: a conceptual framework and case study application. Health Care Manage Rev. 2013;38(2). 10.1097/HMR.0b013e31825e718a 22669050

[47] Wu R, Lo V, Rossos P, Kuziemsky C, O’Leary K, Cafazzo J, Reeves S, Wong B, Morra D (2012). Improving hospital care and collaborative communications for the 21st century: Key recommendations for general internal medicine. Interact J Med Res.

[48] Martínez-García A, Moreno-Conde A, Jódar-Sánchez F, Leal S, Parra P (2013). Sharing clinical decisions for multimorbidity case management using social network and open-source tools. J Biomed Inform.

[49] Rothschilda A, Dietrichb L, Ball M, Wurtzb H, Farish-Huntb H, Cortes-Comererb N (2005). Leveraging systems thinking to design patient-centered clinical documentation systems. Int J Med Inform.

[50] Vawdrey D, Wilcox L, Collins S, Feiner S, Mamykina O, Stein D, Bakken S, Fred M, Stetson P (2011). Awareness of the care team in electronic health records. Applied Clinical Informatics.

[51] Fieschi M (2002). Information technology is changing the way society sees health care delivery. Int J Med Inform.

[52] Cruz-Correia R, Vieira-Marques P, Ferreira A, Almeida F, Wyatt J, Costa-Pereira A (2007). Reviewing the integration of patient data: how systems are evolving in practice to meet patient needs. BMC Med Inform Decis Mak.

[53] Tai-Seale M, Wilson C, Panattoni L, Kohli N, Stone A, Hung D, Chung S (2014). Leveraging electronic health records to develop measurements for processes of care. Health Serv Res.

[54] Hearld L, Alexander J (2012). Patient-centered care and emergency department utilization: a path analysis of the mediating effects of care coordination and delays in care. Med Care Res Rev.

[55] Daniel D, Wagner E, Coleman K, Schaefer J, Austin B, Abrams M, Phillips K, Sugarman J (2013). Assessing progress toward becoming a patient-centered medical home: an assessment tool for practice transformation. Health Serv Res.

[56] Zidel T (2006). A lean guide to transforming healthcare.

[57] Radnor Z, Boaden R (2008). Lean in the public services: panacea or paradox?. Public Money Manag.

[58] Joosten T, Bongers I, Janssen R (2009). The application of Lean to healthcare: issues and observations. Qual Saf Healthc.

[59] Dickson E, Singh S, Cheung D, Wyatt C, Nugent A (2009). Application of lean manufacturing techniques in the emergency department. J Emerg Med.

[60] Mazzocato P, Holden R, Brommels M, Aronsson H, Bäckman U, Elg M, Thor J (2012). How does lean work in emergency care? A case study of a lean-inspired intervention at the Astrid Lindgren Children’s hospital, Stockholm, Sweden. BMC Health Serv Res.

[61] Cima R, Brown M, Hebl J, Moore R, Rogers J, Kollengode A, Amstutz G, Weisbrod C, Narr B, Deschamps C (2011). Use of lean and six sigma methodology to improve operating room efficiency in a high-volume tertiary-care academic medical center. J Am Coll Surg.

[62] Burström L, Letterstål A, Engström M, Berglund A, Enlund M (2014). The patient safety culture as perceived by staff at two different emergency departments before and after introducing a flow-oriented working model with team triage and lean principles: a repeated cross-sectional study. BMC Health Serv Res.

[63] Skeldon S, Simmons A, Hersey K, Finelli A, Jewett M, Zlotta A, Fleshner N. Lean methodology improves efficiency in outpatient academic Uro-oncology clinics. Urology. 2014;83(5). 10.1016/j.urology.2013.11.048 24674117

[64] Laganga L (2011). Lean service operations: reflections and new directions for capacity expansion in outpatient clinics. J Oper Manage.

[65] Martens L, Goode G, Wold J, Beck L, Martin G, Perings C, Stolt P, Baggerman L (2014). Structured syncope care pathways based on lean six sigma methodology optimises resource use with shorter time to diagnosis and increased diagnostic yield. PLoS ONE.

[66] Yousri T, Khan Z, Chakrabarti D, Fernandes R, Wahab K (2011). Lean thinking: Can it improve the outcome of fracture neck of femur patients in a district general hospital?. Injury.

[67] Burgess N, Radnor Z, Davies R (2009). Taxonomy of lean in healthcare: a framework for evaluating activity and impact.

[68] Mcintosh B, Sheppy B, Cohen I (2014). Illusion or delusion – lean management in the health sector. Int J Health Care Qual Assur.

[69] Vermeulen M, Stukel T, Guttmann A, Rowe B, Zwarenstein M, Golden B, Nigam A, Anderson G, Bell R, Schull M (2014). Evaluation of an emergency department lean process improvement program to reduce length of stay. Ann Emerg Med.

[70] Radnor Z, Holweg M, Waring J (2012). Lean in healthcare: the unfilled promise?. Soc Sci Med.

[71] Hines P, Holweg M, Rich N (2004). “Learning to evolve. A review of contemporary lean thinking”. Int J Oper Production Manage.

[72] Rees G (2014). Organisational readiness and Lean Thinking implementation: Findings from three emergency department case studies in New Zealand. Health Serv Manage Res.

[73] Waring J, Bishop S (2010). Lean healthcare: Rhetoric, ritual and resistance. Soc Sci Med.

[74] Lega F (2008). The rise and fall(acy) of clinical directorates in Italy. Health Policy.

[75] Bartunek J, Rousseau D, Rudolph J, Depalma J (2006). On the receiving end: Sense-making, emotion, and assessment of an organizational change initiated by others. J Appl Behav Sci.

[76] Herold D, Fedor D, Caldwell S (2007). Beyond change management: a multilevel investigation of contextual and personal influences on employees’ commitment to change. J Appl Psychol.

