# Supplementary material for: Exploring “patient-centered” hospitals: a systematic review to understand change
Source: BMC Health Serv Res. 2017 May 22;17:364. doi: 10.1186/s12913-017-2306-0 (PMC5439229; doi:10.1186/s12913-017-2306-0)
Supplement: Additional file 1: — Search strategy and filters. (DOCX 12 kb) [file 12913_2017_2306_MOESM1_ESM.docx]

Search strategy: “(Hospital OR healthcare) AND (change management OR organizational model)”

Languages: English, Italian

Filters: web of science categories: health policy services

research areas: health care sciences services
